# Fitness-maximizers employ pessimistic probability weighting for decisions under risk

**DOI:** 10.1017/ehs.2020.28

**Published:** 2020-06-01

**Authors:** Michael Holton Price, James Holland Jones

**Affiliations:** 1 Santa Fe Institute, Santa Fe, California, USA; 2 Department of Earth System Science, Stanford University, Stanford, California, USA; 3 Woods Institute for the Environment, Stanford University, Stanford, California, USA; 4 Department of Life Sciences, Imperial College, London, UK

**Keywords:** Utility, risk, behavioural economics, evolutionary demography, fitness

## Abstract

The standard theory of rationality posits that agents order preferences according to average utilities associated with different choices. Expected utility theory has repeatedly failed as a predictive theory, as reflected in a growing literature in behavioural economics. Evolutionary theorists have suggested that seemingly irrational behaviours in contemporary contexts may have once served important functions, but existing work linking fitness and choice has not adequately addressed the challenges of constructing an evolutionary theory of decision making. In particular, fitness itself is not a reasonable metric for decision making since its timescale exceeds the lifespan of the decision-maker. Consequently, organisms use proximate systems that work on appropriate timescales and are amenable to feedback and learning. We develop an evolutionary principal–agent model in which individuals utilize a set of proximal choice variables to account for the non-linear dependence of these variables on consumption. While this is insufficient to maximize fitness in the presence of environmental stochasticity, maximum fitness can be achieved by adopting pessimistic probability weightings compatible with the rank-dependent expected utility family of choice models. In particular, pessimistic probability weighting emerges naturally in an evolutionary framework because of extreme intolerance to zeros in multiplicative growth processes.

## Graphical abstract

**Figure:**
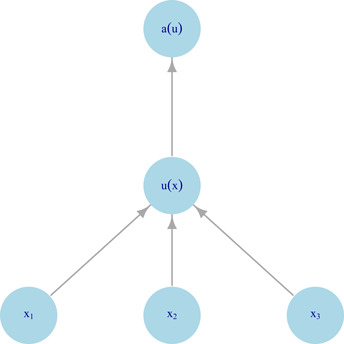

**Media summary:** Fitness-maximizers employ pessimistic probability weighting for decisions under risk.

## 1. Introduction

A herder in in the arid north of Kenya has to make decisions about the composition of his herd (Mace & Houston, 1989; Mace, 1990, 1993). Should he restrict his herd to small, high-productivity goats or, when he gets enough goats, should he trade them for larger, slower-breeding camels? The growth rate of goat herds, even accounting for their increased susceptibility to high mortality during drought years, is three times that of camels and the expected energetic payoff to keeping goats – more precisely, the instantaneous rate at which wealth accumulates – is therefore substantially higher than a mixed herd of goats and camels. However, pastoralists in the region favour mixed herds and will even increase the fraction of camels dramatically above a critical wealth threshold (Mace &Houston, 1989; Mace, 1990). Are these herders engaging in irrational economic decision making by under-valuing the payoff of an all-goat herd? While the expected return is lower for a mixed herd of predominantly camels, households pursuing this strategy have a much higher probability of long-term persistence, nearly 70%, compared with a 0.4% probability of persistence for the goats-only strategy. Why is this so?

In part, mixed herding diversifies risk. More importantly, however, the growth of these herds, like any biological growth process, is multiplicative and the rate of increase is stochastic. Furthermore, the herder faces more than just a decision about long-term wealth accumulation, a well-studied topic in finance (e.g. Karatzas & Shreve, 1998) and economics (e.g. Peters & Gell-Mann, 2016). Rather, herd products and income from the herd provide each household's primary yearly consumption, and consequently there are two processes that matter to the herder: herd growth and household consumption. We might better interpret the herder's decision as not about herd management, *per se*, but rather about household consumption through time, for which herd dynamics can lead to very uneven yearly consumption that cannot be fully accounted for by available smoothing mechanisms.

Like growth of the herd, household survival can be thought of as a stochastic gamble in which the survival probability is a function of household consumption. For such a multiplicative process, a severe shortfall in any time period that leads to a low survival probability can never be compensated for by high survival in other time periods (even a large number of other time periods). A virtually identical perspective arises when considering the evolutionary fitness of individuals because, as we will describe and formalize, fitness is determined by stochastic, multiplicative processes; most obviously, individual survival through time is a component of fitness, and like household survival, individual survival is a binary outcome variable with differing probabilities for each time period. In this article, we explore what this means for the evolution of organisms’ decision making.

Our challenge is to account for how selection might construct a decision-making system that is responsive to fitness-relevant decisions in real, variable environments. The first core contribution of this paper is our suggestion of a hierarchical framework for evolutionary decision making that arises naturally from Mayr's proximate/ultimate distinction (Mayr, 1961). We then employ the logic of an evolutionary principal–agent problem, an idea we adopt from Binmore (1994), to link proximate and ultimate ends. This framework reflects the limitations natural selection faces in imparting fitness-optimizing behaviours to organisms. Other authors have suggested that natural selection addresses these limitations by imparting proximate preferences to agents. However, we add that natural selection may have separately, or in tandem, influenced more than one component of human decision making. Much like the utility function in expected utility theory, proximate currencies can be used to map the consequences of specific decisions onto evolutionarily salient proximate variables such as fertility and survival. The hierarchical model we propose possesses proximate value functions that map consumption onto proximate variables, while probability weighting fixes a mismatch that arises from the enforced inability of these value functions to respond to time-dependent environmental uncertainty.

Our second core contribution arises from applying this framework and understanding the consequences of selection for both the value functions mapping consumption to proximate currencies and the probability weighting that then links these to fitness. It has long been recognized in evolutionary biology that stochastic fluctuations in single-period growth rates lower the long-term growth rate below what would be achieved from deterministic growth at the mean rate each period (Haldane & Jayakar, 1963; Lewontin & Cohen, 1969; Tuljapurkar, 1982). In our hierarchical framework, we limit the role of value functions to only map consumption onto proximate outcomes such as age-specific fertility and mortality (we do allow that the mapping itself can depend on environmental or social contexts). Hence, they can play no role in addressing the long-term lowering of fitness exacted by environmental stochasticity. This is where the probability weighting contributes to the model. The optimal level of weighting does depend on the exact universe the organism occupies, which sets the gambles the organism faces and how those gambles vary with time. We show that, regardless of these details, the optimal probability weighting involves pessimistic probability weighting. This result is of fundamental theoretical importance because it links evolutionary theory with important work in economics. Pessimistic probability weighting of unlikely, negative outcomes is a core aspect two of the most successful behavioural economic models, rank-dependent expected utility theory and cumulative prospect theory, both of which recover expected utility theory as a special case. We thus offer a grounded evolutionary explanation for one of the cornerstones of modern behavioural economics.

Because we anticipate a diverse readership for this article, we have provided substantial background material, especially in Sections 2 and 5. Section 2 summarizes pertinent economic ideas, notably expected utility theory and alternatives to it such as rank-dependent expected utility theory. Section 3 frames our hierarchical model with respect to recent literature on the evolution of economic preferences, describes the evolutionary principal–agent problem and summarizes our hierarchical model. Section 4 gives a high-level, verbal outline of our model. Section 5 describes the evolutionary component of our model, stochastic age-structured population models (the economic model, rank-dependent expected utility theory, is described in Section 2). Section 6 brings together the preceding material to establish our core result on pessimism. Section 7 discusses our results.

## 2. Expected utility theory and its alternatives

Organisms, humans included, must make decisions about foraging, reproductive, social and political behaviours that have consequences for proximate outcomes such as satiety, income, wealth, happiness, sexual satisfaction and well-being. These decisions also have consequences for ultimate outcomes such as fitness and, consequently, there are strong expectations that selection acting on differential fitness will shape the decision-making system. The rational-choice tradition suggests that individuals make decisions that maximize some quantity typically denoted by the catch-all term ‘utility’. In the presence of variability in outcomes, the objective function for maximization is the expectation of utility. In particular, a set of possible payoffs (i.e. a ‘lottery’) 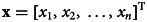 with associated probabilities 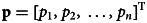 is preferred to some other lottery 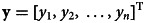 with probabilities 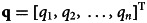 if some preference functional (Machina, 1982) that associates payoffs with probabilities 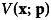 is greater than that associated with 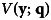: 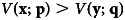. Here and elsewhere vectors (bolded) are column vectors by default and ^T^ indicates a transpose. A preference functional thus contains two inputs: (1) a measure of the value of outcomes (i.e. utilities associated with outcomes); and (2) weights for these outcomes. In the absence of other information, a reasonable value function is linear in the probabilities and utilities of distinct outcomes,1
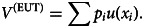


This linear specification yields an expectation over utility and the theory that uses maximization of expected utility is known as expected utility theory (EUT). This theory was first formalized in its modern form by von Neumann and Morgenstern (1947) and further axiomatized by Savage (1954). Expected utility, combined with exponential time discounting of delayed outcomes, represents the canonical economic model of decision making (Samuelson, 1937; von Neumann & Morgenstern, 1947; Machina, 1982; Starmer, 2000; Frederick et al., 2002). The EUT approach has been very fruitful for economics, political science and other behavioural sciences. Like many economic theories it is axiomatic and the fundamental axioms that underlie EUT (completeness, transitivity, continuity and independence) are sensible requirements that ensure that preferences are consistent (von Neumann & Morgenstern, 1947; Savage, 1954). However, an enormous literature has developed showing that people violate the axioms underlying EUT in both experimental and naturalistic contexts. Some examples include the common consequence effect (Allais paradox), the common ratio effect (Allais, 1953), ambiguity aversion (Ellsberg, 1961), preference reversals between gambles represented as bids vs. choices (Lichtenstein & Slovic, 1971), the incommensurability of risk-sensitive behaviour for high- vs. low-stakes gambles (Rabin, 2000), overconfidence (Heller, 2014) and abundant evidence that framing and reference points induce departures from canonically predicted behaviour (Kahneman & Tversky, 1979; Tversky & Kahneman, 1992; Loewenstein & Prelec, 1992). Violations of the stationarity of time preferences, another canonical assumption although not the focus of this article, include the common difference effect and the absolute magnitude effect (Loewenstein & Prelec, 1992; Frederick et al., 2002).

One consequence of these empirical observations is that they spurred theorists to develop new choice models that could accommodate the empirical findings. Starmer (2000) divides these efforts into conventional and non-conventional. Regarding the conventional strategy, Starmer (2000, p. 338) writes, ‘The general spirit … is to seek “well behaved” theories of preferences consistent with observed violations of independence’. In this spirit, Starmer defines conventional theories as those that maintain the completeness, transitivity and continuity axioms, but rather than maintaining the independence axiom insist on monotonicity, ‘the property that stochastically dominating prospects are preferred to prospects which they dominate’ (Starmer, 2000, p. 335). First-order stochastic dominance occurs when the cumulative distribution function (or cumulative mass function for a discrete distribution) of a gamble is at least equal to that of another gamble over its entire domain (Quiggin, 1993, p. 77). It is common to require the cumulative distribution function to be strictly greater than that of the other gamble on at least part of its domain (Wakker, 2010, p. 65).

The reason Starmer uses independence as the criterion to distinguish conventional from non-conventional theories is that its violation was (and almost certainly is) seen as the primary challenge to EUT arising from behavioural economics experiments. The independence axiom asserts that adding a common outcome to two prospects should not change the preference ordering of the prospects. Perhaps the most notable behavioural anomaly is the Allais paradox (Mongin, 2019, for a recent review), or common consequence effect, which uncovers a violation of the independence assumption. Quiggin (1993, p. 30) writes: ‘The Allais problem is the *pons asinorum* of theories of choice under uncertainty. Almost all of the many authors who have introduced new models of choice under uncertainty in the last 10 years [i.e. alternatives to expected utility theory] have included a demonstration that the model is consistent with the behaviour revealed in this problem’.

Machina (1981, 1987) noted there is nothing inevitable about expected utility serving as the objective function for preference ordering and suggested that nonlinear functions mapping proximate values and probabilities might account for these types of systematic departures from the predictions of EUT. An important class of non-linear models that can accommodate the Allais paradox does so via subjective probability weights. In the simplest formulation of probability weighting, the decision weights are a direct function of the probability of each individual outcome,2
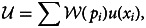
where 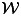 is the weighting function and *p*_*i*_ is the probability of outcome *i*. Preson and Baratta (1948) may have been the first to suggest the use of subjective probability weights in this way. They showed that when individuals could competitively bid on lotteries with uncertain outcomes they systematically over-weighted low probabilities (up to about *p* = 0.1) and under-weighted high probabilities. This straightforward method of probability weighting was adopted in the original formulation of prospect theory (PT; Kahneman & Tversky, 1979), an early, heuristic-based alternative to EUT that gained widespread attention. However, it was quickly pointed out that PT and other theories that simplistically implement probability weighting violate monotonicity (Wakker, 2010, p. 153). An ad hoc fix of the PT violation of stochastic dominance was incorporated via an editing process that occurred before the probability weighting occurred. In addition to lacking parsimony, this editing process can induce intransitivity in pairwise choices (Quiggin, 1982). The appeal of PT was its selective incorporation of the more desirable features of EUT, while nevertheless rejecting the independence axiom, so that the Allais paradox and other behavioural violations could be accommodated, but a model that leads to the favouring of stochastically-dominated choices or intransitivity was clearly unacceptable.

The publication and response to PT spurred work to develop alternatives, including axiomatic, conventional theories (*sensu* Starmer) that maintained EUT's less controversial axioms (completeness, transitivity, and continuity) while simultaneously avoiding the violation of stochastic dominance exhibited by PT in its unedited form. The culmination of this work was the simultaneous and independent discovery of RDEUT by at least three scholars (Quiggin, 1982; Schmeidler, 1989; Yaari, 1987). RDEUT avoids violations of monotonicity by re-weighting probabilities in a quite specific manner. Assume that the probabilities of some prospect are sorted from most desirable to least desirable according to the values of their corresponding outcomes (*x*_1_ ≥ *x*_2_ ≥ *x*_3_ ≥ ⋅ ⋅ ⋅ ). Next, define the cumulative probability, or rank (Wakker, 2010), as3
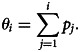
where *θ*_*i*_ is the probability of receiving an outcome that is as good as or better than *i*. The probability weighting on outcome *i* is given by4
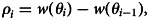
where *w* is the weighting function defined on ranks and we assume that *θ*_0_ = 0. In equation (4), *w* is used rather than 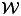 to emphasize that *w* is defined on probability ranks, whereas 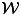 is defined on probabilities; *w* maps the interval [0, 1] onto itself, must be an increasing function of *θ*, and must satisfy *w*(0) = 0 and *w*(1) = 1. The rank-dependent expected utility is5
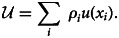


**Figure 1.**
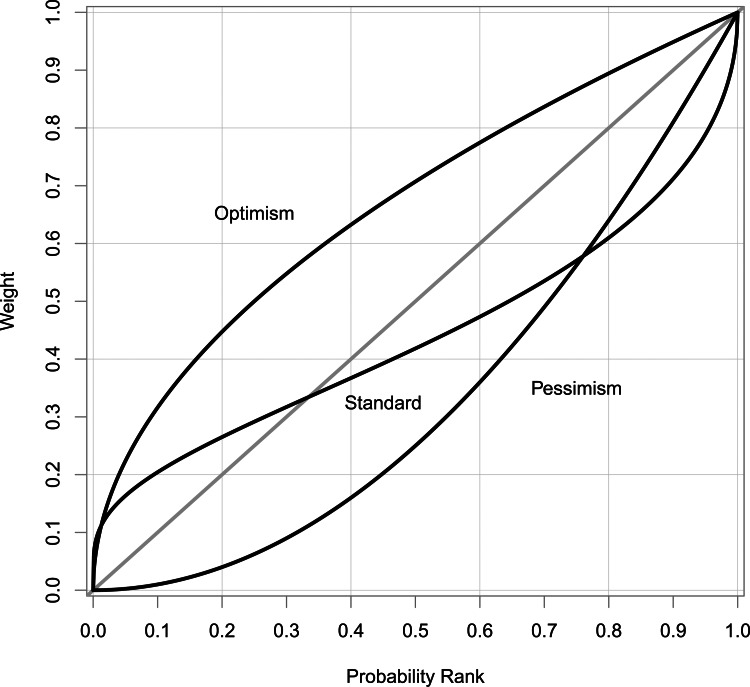
Probability weighting functions for the RDEUT model. The top curve exhibits optimism over its entire domain. The bottom curve exhibits pessimism. The middle curve is a standard curve adopted in much of the recent literature; it combines pessimism with likelihood sensitivity.

Figure 1 plots three different probability weighting functions. The middle curve is a standard form that exhibits two suggested biases in the psychophysics of probability distortions: likelihood sensitivity and pessimism (Gonzalez and Wu, 1999; Wakker, 2010). Likelihood sensitivity occurs because differences near the endpoints of the probability scale (0 and 1) loom larger than differences in the interior of the interval. Conversely, differences in the middle of the scale loom smaller, so that the difference between 0 and 1% is psychologically much more salient than the difference between 60 and 61%. To account for diminished likelihood sensitivity for intermediate probabilities, the probability weighting function must have an inverse-S shape so that the centre is more flat than the endpoints.

Pessimism involves the under-weighting of desirable outcomes relative to their probability of occurrence, and leads to an overall lowering of the weighting function. Two types of pessimism can be defined relative to the probability weighting function, *w*(*θ*): regular pessimism and strong pessimism (Quiggin, 1993). Regular pessimism (or simply pessimism) occurs where *w* is below the identity line *w* = *θ*, whereas optimism occurs where *w* is above the identity line. Strong pessimism occurs where *w* is convex (second derivative greater than zero), whereas strong optimism occurs where *w* is concave (second derivative less than zero). For many functional forms of *w*, regular pessimism and strong pessimism occur on the same or nearly the same intervals (and similarly for optimism).

RDEUT accounts for the Allais paradox through pessimistic probability weighting. Segal (1987), for example, has formulated a general statement of the Allais paradox using arbitrary payoffs and non-discrete probability distributions, and shown that RDEUT with a probability weighting function *w* exhibiting strong pessimism on its entire domain (i.e. the unit interval) can accommodate any and all formulations of the generalized Allais paradox. However, only a subset of the generalized formulations are encountered in daily life or economic choice experiments, so less restrictive probability weighting functions can accommodate the empirically relevant cases, although some pessimism is still a necessary element. For example, as already discussed, pessimism is one of the two primary components of the standard probability weighting curve (the other being likelihood sensitivity), which exhibits pessimism over only part of its domai *n*. Consequently, we utilize regular pessimism as opposed to strong pessimism as the relevant definition of pessimism. A crucial result proven by Quiggin (1993, chapter 6) follows from this assumption:

Theorem 1. *If an RDEUT decision maker (agent) possesses a function u*(⋅) *that is increasing and concave, the following are equivalent,*

- *the agent exhibits regular pessimism, w*(*θ*) ≤ *θ*.
- *the agent ascribes a lower value to the RDEUT than the EUT valuation, V*^(*RDT*)^ ≤ *V*^(*EUT*)^.

When it was realized that RDEUT avoided violations of stochastic dominance and could accommodate the Allais paradox, PT was reformulated to include RDEUT, becoming cumulative prospect theory (CPT; Tversky & Kahneman, 1992). Consequently, RDEUT, either in its own right or as a component of CPT, is one of the most important components of contemporary work to generalize EUT (Starmer, 2000; Wakker, 2010).

Having summarized some of the pertinent economic ideas and models, we can now to turn to the central focus of the article. What mechanistic or functional basis might nonlinear preference functionals have? A reasonable working hypothesis is that their origins are found in evolutionary history. Indeed, there is a thread of evolutionary logic that runs through parts of the behavioural-economic literature. For example, an interpretation of Kahneman & Tversky's dual-process model, as articulated by Kahneman (2003, p. 697), suggests that ‘intuitive judgments occupy a position – perhaps corresponding to evolutionary history – between the automatic operations of perception and the deliberate operations of reasoning’. Various well-known decision biases – particularly loss-aversion – have been interpreted in light of evolutionary success (Haselton & Nettle, 2006; McDermott et al., 2008). Aside from this, there is a small but growing literature, mostly within economics, that seeks to model the evolution of human economic preferences (Rogers, 1994; Robson, 1996; Robson & Samuelson, 2009).

## 3. Natural selection, preferences and the evolutionary principal–agent problem

We are interested in economic decisions in the broadest sense. At the basic level, organisms are making decisions over what can be thought of as different lotteries. For example, should a herder focus on one animal or create a mixed herd? Should a forager hunt for sand monitor lizards or hill kangaroo (Jones et al., 2013)? Should a peasant farmer intensify cultivation of a nearby garden plot or spread effort across two geographically distinct plots (Norman, 1974)? Should a woman wean her infant and have another baby or continue nursing and delay reproduction (Hobcraft et al., 1983; Jones and Bliege Bird, 2014)? Should an individual buy or rent a home (Shelton, 1968). In these examples, each lottery yields different payoffs probabilistically. All of these examples could have a clear impact on fitness, but it is extremely unlikely that any conscious fitness-maximization goal plays significantly into any of the decision makers’ choices. Instead, their decisions are shaped by preferences over a variety of proximate currencies like hunger/satiety, feelings of security or feelings of love and responsibility for children. Samuelson and Swinkles (2006) raise the important question, given the evolutionary mandate to successfully leave descendants, why do people have preferences for anything but fitness?

### Why have preferences for proximate quantities?

In the substantial literature that addresses the question of why people have preferences for proximate currencies rather than fitness itself (Rogers, 1994; Binmore, 1994; Robson & Samuelson, 2011; Glimcher, 2016), three inter-related factors loom largest. First, natural selection operates on timescales that are longer than the lifespans of the organisms whose behaviour it shapes and, furthermore, selection is a stochastic, undirected process. Second, organisms regularly encounter novel situations for which natural selection is unable to directly specify behaviours. Third, the types of solutions that might emerge via natural selection to address the first two factors are constrained by trade-offs imposed by the cost of gathering and processing information. These observations can be accommodated within a single analytic framework by utilizing the economic concept of the principal–agent problem (Binmore, 1994), which will allow us to address the question of why organisms have preferences defined over proximate currencies, rather than the ultimate currency of fitness.

Consider a principal that possesses certain goals that it is unable to achieve unless it acts through agents over which it has only indirect control (Binmore, 1994). Natural selection is the ultimate arbiter of which biological entities remain and increase in a population. While the process of natural selection lacks agency, it is nonetheless useful to consider the outcomes of selection as having been designed (Williams, 1966). It is in this sense that natural selection can be thought of as a principal with a goal of maximization of fitness. However, there are clear limitations in the ability of selection to achieve a solution, as enumerated above. Owing to the obvious lack of direct control, selection shapes cognitive mechanisms which are, on the whole, consistent with fitness maximization. As noted by Binmore (1994), these collective proximate cognitive mechanisms can be thought of as the agent in the evolutionary principal–agent problem, wherein the principal (selection) ‘seeks to design an incentive scheme that minimizes the distortions resulting from having to delegate to the agents’ (Binmore, 1994, p. 151). The extent to which selection minimizes these distortions for a given organism in a given setting depends on the structure and strengths of the constraints it faces. A virtually identical perspective underlies the so-called ‘indirect approach’, in which the utility function is defined on proximate goods or outcomes, and the utility function in turn determines the success of an organism (Güth & Yaari, 1992; Güth, 1995; Dekel et al., 2007).

**Figure 2.**
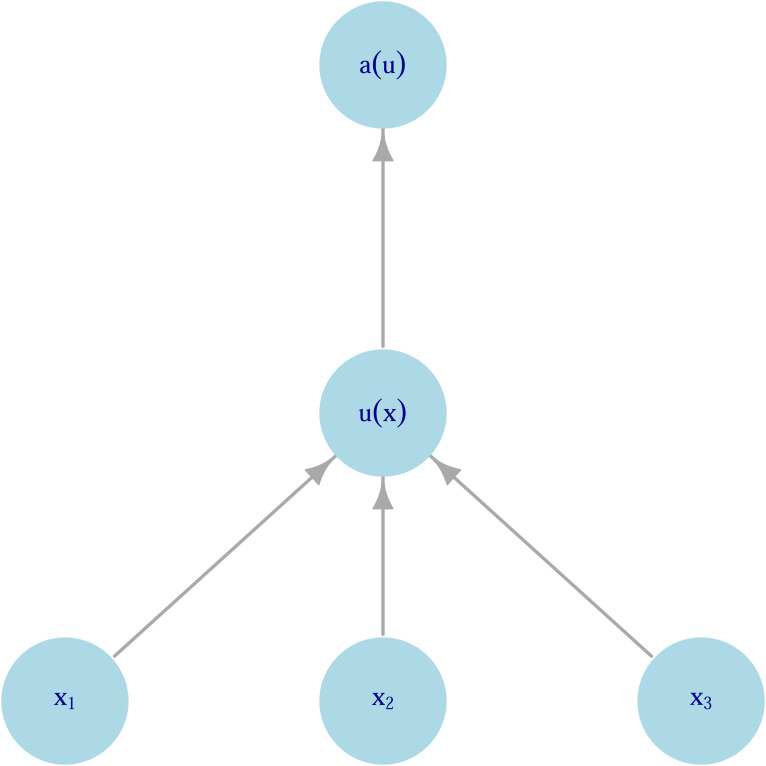
Hierarchical model of decision making as a principal–agent problem. Input variables (**x**_**i**_) contribute to intermediate utility (*u*(*x*)), which itself contributes to fitness (*a*(*u*)).

Figure 2 encapsulates the conceptual model that emerges from the principal–agent framework. At the bottom level, an individual is choosing between lotteries that influence the outcomes 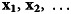. The outcomes of these lotteries contribute to utilities, *u*_*j*_, at the next level. These lotteries can be thought of either as motivational systems such as satiety, sexual gratification or happiness (akin to the classical notion of utility) or as proximate determinants of fitness such as infant survival or total fertility. What links these two different notions of ‘utility’ is that they work on a timescale where the organism can use feedback from outcomes to change its preferences and therefore decision making. These proximal utilities then contribute ultimately to fitness.

## 4. Model outline

The preceding section describes a conceptual framework for understanding the evolution of economic preferences. The main conclusion is that the evolutionary principal–agent problem implies the existence of proximate decision-making mechanisms within a hierarchical framework. In this section, we describe how to implement a formal model in accordance with this conceptual framework. In so doing, we provide a high-level summary of the actual model described below upon which our results and conclusions are based. Three elements are needed to apply the conceptual framework encapsulated by Figure 2: (1) an evolutionary model that defines how fitness depends on some determinants of fitness; (2) an economic model of decision making; and (3) a mechanism to link the evolutionary and economic models and thus provide novel predictions or insight into behaviour.

### Evolutionary model

The evolutionary formalism we use is stochastic age-structured life history theory as described by Tuljapurkar (1982). We assume a randomly mating, diploid population. Different phenotypes make different consumption decisions when faced with risk and environmental uncertainty. These differing consumption streams lead to different realizations of age-specific survival and fertility and thus to different growth profiles for the phenotypes. Tuljapurkar (1982) shows that the rate of exponential growth (logarithm of the growth rate) of a phenotype, *a*, governs invade-ability (formalized below). In terms of Figure 2, the rate of exponential growth is the measure of fitness whereas age-specific survival and fertility – which change through time – are the determinants of fitness. The core result is equation (13), and readers already familiar with this result could skip the other material in the section.

### Economic model

The economic formalism we use is RDEUT, which is summarized above in Section 2. The key result is Theorem 1.

### Linking the models

To link the models, we assume that the determinants of fitness in the evolutionary model (age-specific survival and fertility) can be associated with the utility function in EUT. That is, at least to first order, an organism makes proximate decisions based on the expected values of the determinants of fitness. Other assumptions can be made to link the models, but we consider this an eminently sensible assumption to make given the limitations implied by the evolutionary principal–agent problem. It seems much easier for an organism to reason about the mean number of offspring a given strategy will yield (perhaps with a time discount factor applied on the timing of reproduction) than for an organism to reason about the ultimate fitness consequences of a given strategy.

However, we will show that averaging survival or fertility is systematically ‘wrong’ – i.e. leads to lower fitness – when stochastic uncertainty in achieved outcomes is accounted for. Specifically, the true fitness of a strategy that involves stochastic uncertainty is lower than that implied by the corresponding mean strategy that accounts for only mean survival and fertility. This is precisely the condition for pessimism in RDEUT and implies that an organism can ‘correct’ its expected utility bias by re-weighting the probabilities of a gamble as in RDEUT.

## 5. Stochastic, age-structured population matrices

To model the population dynamics and genetics, we utilize the formalism described by Tuljapurkar (1982). We assume a diploid locus with alleles 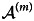 (*m* = 1, 2, 3, …), random mating and a fixed sex-ratio, and ignore sex-differences. The genotypes 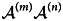 code behavioural strategies that interact with the environment to determine an individual's life-history traits in each time period. Tuljapurkar (1982) derives two essential results. First, he shows that the rate of exponential growth of 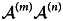 governs invade-ability. That is, a population of homozygotes with allele 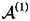 is resistant to invasion by allele 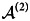 if *a*^(11)^ > *a*^(12)^, where *a*^(*mn*)^ is the rate of exponential growth achieved by genotype 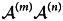. Second, Tuljapurkar provides an analytical formula for *a* when the variance in life history traits is small. The fundamental tool on which these results rely is the Leslie matrix; we introduce the Leslie matrix in the next section, then describe how stochastic uncertainty can be added.

### Life history traits

Consider an age-structured population where each age class *j* accounts for individuals between *y*_*j*_ = *j* Δ*y* and *y*_*j*+1_ = (*j* + 1) Δ*y* years of age. The key demographic traits that govern the population's dynamics are the age-specific fertility, *F*_*j*_, and the age-specific survival probabilities, *P*_*j*_. *F*_*j*_ is the mean number of offspring that an individual in age class *j* contributes to the juvenile age class (*j* = 1) going from time *t*_*n*_ to time *t*_*n*+1_ = *t*_*n*_ + Δ*y*, where *n* indexes time steps. *P*_*j*_ is the probability that an individual will survive from age class *j* to age class *j* + 1. The Leslie matrix, 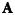, has the age-specific fertilities on the first row and the age-specific survivals on the sub-diagonal,6
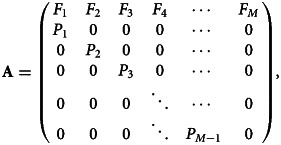
where *M* is the number of age classes. The Leslie matrix projects the population vector 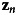 one time step into the future, 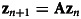. We assume that the Leslie matrix is irreducible and primitive (Caswell, 2001). Given these assumptions, there exists a unique dominant eigenvalue *λ* that is the per-period growth factor of the population. In the absence of stochastic fluctuations of the Leslie matrix elements, *a* = log *λ* (stochasticity is considered below). Hence, a population of homozygotes with genotypes 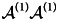 is resistant to invasion by allele 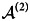 if *λ*^(11)^ > *λ*^(12)^. Associated with *λ* are the dominant left and right eigenvectors of 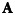, which we represent by ***v*** and ***ω***, respectively. ***v*** is the vector of age-specific reproductive values and ***ω*** is the stable age distribution vector.

### Stochastic uncertainty

We now account for stochastic uncertainty in the Leslie matrix elements. The crucial idea is that the Leslie matrix is not fixed from one time step to another, but instead depends on the changing state of the world. Following Tuljapurkar (1982), consider an environmental sequence 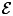 with an associated sequence of Leslie matrices 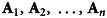. Let7
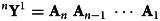
represent the product matrix which governs population growth to time period *n*, and let *λ*_*n*_, ***ω***_*n*_, and ***v***_*n*_ represent, respectively, the dominant eigenvalue, corresponding right eigenvector and corresponding left eigenvector of 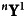. Let 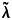, 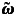, and 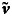 represent the corresponding quantities for the mean Leslie matrix. Without loss of generality, we assume that ***ω***_*n*_ and ***v***_*n*_ are normalized to 1. Weak ergodicity guarantees certain results. First, it guarantees that8
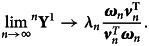


Second, it guarantees the convergence of age structure to a stable age structure for any non-negative and non-zero starting vector 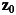. To formalize this, let9
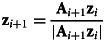
be the normalized age structure at time period *n*, where the normalization is 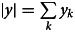. Then10
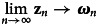


An analogous result holds for 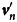 with left multiplication. The rate of exponential growth is11
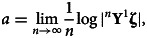
where **ζ** is an arbitrary initial age structure. Stochasticity lowers the rate of exponential growth compared with a non-stochastic reference Leslie matrix with the same mean life history traits. Effectively, this is because growth is a multiplicative process and the geometric mean is always less than the arithmetic mean (Lewontin & Cohen, 1969). Tuljapurkar (1982) derives a useful ‘small noise approximation’ for *a* assuming small fluctuations of the Leslie matrix elements relative to the mean values,12
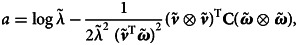
where 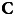 is the covariance matrix of the mean covariance matrix 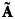, ⊗ is the Kronecker product, and the serial auto-correlation term in Tuljapurkar's approximation is not included. If only one Leslie matrix element is considered, equation (12) simplifies to13
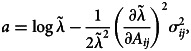
where 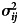 is the stochastic variance associated with matrix element *A*_*ij*_. Equation (13) is the key evolutionary result. Caswell (2010) provides a formula for the partial derivatives of *λ* with respect to the matrix elements,
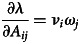


### Accommodating the hierarchical framework

The elements of the Leslie matrix, *A*_*ij*_, are the determinants of fitness at the intermediate level of Figure 2 (in this section, we do not explicitly show the time dependence of these matrix elements). We assume that they depend on a valuable but limited resource that can be traded off between fertility and survival across an individual's life cycle. More precisely, we assume that each Leslie matrix element *A*_*ij*_ is an increasing function of the consumption *x*_*ij*_ allocated to it with a negative second derivative (the latter condition ensures that weak pessimism is equivalent to the RDEUT sum being less than or equal to the EUT sum (Quiggin, 1993, chapter 6). *x*_*ij*_ is at the bottom level of Figure 2. For the reasons outlined in Section 3, we limit the role of proximate preferences in our model to accounting for the functional dependence of the *A*_*ij*_ on the *x*_*ij*_, including how these mappings may depend on social or ecological context. This leaves a fitness mismatch that cannot be accommodated by proximate preferences since environmental stochasticity, which lowers the time-averaged rate of exponential growth, is unaccounted for. In the next section, we show that accounting for the effect of environmental stochasticity using probability weights implies pessimistic probability weighting.

## 6. Pessimism

In this section, we show that organisms that face uncertainty – arising, e.g. from the interaction of environmental fluctuations interacting with strategic choices – will act as pessimistic decision makers *sensu* RDEUT. Let *s* index strategies an agent can choose and let *k* index distinct outcomes (for example, achieved survival or fertility in a given time period). The agent faces uncertainty in choosing the strategy since the state of the world is not known when the strategy is chosen, but the agent does know the probability 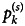 that each outcome will occur given strategy *s*. For notational convenience, we assume the outcome variable is age-specific fertility in some age class. For outcome *k*, the achieved fertility is *f*_*k*_; we use a lower case *f* to distinguish this fertility from the age-specific fertilities of the Leslie matrix in equation 6. The mean fertility for strategy *s* is14
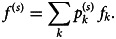


Table 1 illustrates this model for a simple case with three possible strategies and five possible outcomes. What makes these three strategies interesting is that they offer the same expected utility (i.e. fertility) but, as we show next, different evolutionary fitnesses. In particular, strategy *s* = 1 offers the highest fitness because it has the lowest variance and strategy *s* = 3 offers the lowest fitness because it has the highest variance. The gambles we discuss in this section all depend implicitly on underlying consumption levels that determine fertility. However, the details of the dependence do not impact the results so for simplicity we choose not to explicitly show them. Let 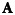 represent a Leslie matrix in which all elements are fixed except for one stochastic fertility term, 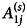, which equals *f*_*k*_ with probability 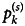 as described above and summarized in Table 1. In equation (13), the second term is always negative. The effect of stochastic uncertainty, therefore, is to reduce *a* if the mean Leslie matrix is held constant. That is, 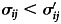 implies 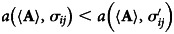, and vice versa. Strategy *s* = 1 in Table 1 is preferred to *s* = 2 and *s* = 2 is preferred to *s* = 3 since variance increases from *s* = 1 to *s* = 3.

**Table 1.** Three strategies with the same expected utility (fertility) but different evolutionary fitnesses; *s* = 1 is preferred to *s* = 2 is preferred to *s* = 3 since variance increases from *s* = 1 to *s* = 3. The first five rows summarize the outcomes for each achieved fertility level, with the first column giving the index *k*, the second column the fertility for each outcome, *f*_*k*_, and the remaining columns the probabilities of each outcome given the chosen strategy *s*. The last two rows summarize, for each strategy *s*, the mean and standard deviation for fertility

	*f*_*k*_	*s* = 1	*s* = 2	*s* = 3
*k* = 1	0.5			0.5
*k* = 2	1.0		0.5	
*k* = 3	1.5	1.0		
*k* = 4	2.0		0.5	
*k* = 5	2.5			0.5
*f*^(*s*)^		1.5	1.5	1.5
*σ*^(*s*)^		0	0.5	1

The stage is now set to incorporate the economic theory and show that stochastic uncertainty induces deviations from expected utility maximization consistent with pessimistic subjective probability weighting. An expectation maximizer will evaluate strategies solely by the mean fertility, *f*^(*s*)^, which is equivalent to writing 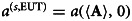, where *a*^(*s*,EUT)^ is the expected utility valuation of the fitness. Symbolically, we can write15
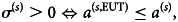
where ⇔ indicates that the left-hand side implies the right-hand side and the implication is in both directions. This result is pertinent to RDEUT since the definition of pessimism in RDEUT is that a re-weighted lottery is valued as less than its expected-utility value (Theorem 1). For a concave utility function, this is equivalent to assuming that *w*(*p*) ≤ *p* for all *p* (Theorem 1). Symbolically, we can write16
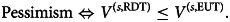


If we assume that natural selection (the principal) has imparted subjective probability weighting to the agent in order to ‘fix’ the optimism of the EUT decision rule, we can posit, by comparing equation (15) with equation (16), that natural selection should instill its agents with pessimistic subjective probability weights. It is worth emphasizing that the example in Table 1 is merely for illustration. Equations (14)–(16) apply generally given equation (13).

## 7. Discussion

The key scientific finding of this article is the derivation of RDEUT-like pessimistic decision weighting from evolutionary first principles. An explanation for pessimistic probability weighting emerges naturally from coupling utility-maximizing decision making and fitness maximization. This pessimism arises from a profound intolerance for zeros in key evolutionary parameters which, in turn, arises from a fundamental difference between economic utility and fitness. As we have previously observed, cumulative expected utility is additive across time periods, whereas fitness and key components of fitness, such as survival, are multiplicative across time, a point made for models of the ‘survival’ of firms by Radner (1998). Consequently, additive decision metrics such as EUT do not necessarily lead to optimal behaviour from a fitness standpoint, and can even lead to catastrophic outcomes. This explains the apparent violations of EU optimization observed in subsistence populations such as the herders discussed in the introduction.

There is, in fact, a striking correspondence between the evolutionary intolerance for zeros and the theoretical foundations of RDEUT. John Quiggin, in describing the mentality with which he approached the derivation of RDEUT, writes, ‘The crucial idea was that the overweighting of small probabilities proposed by Handa and others should be applied only to low probability extreme outcomes, and not to low probability intermediate outcomes’ (Quiggin, 1993, p. 56). Since low probability extreme outcomes are the key factor driving both the evolutionary intolerance for zeros and the development of RDEU, it may come as no surprise that our evolutionary model predicts the biasing of probability weights for survival and fertility (i.e. utility) in a manner consistent with RDEUT. Aversion to zeros has been used in population biology to explain a range of life-history phenomena which are not favoured under standard non-stochastic models such as the regular production of clutches smaller than the most productive clutch (Boyce & Perrins, 1987), delayed reproduction (Tuljapurkar, 1990) and iteroparity (Orzack & Tuljapurkar, 1989).

Our results suggest that evolution will strongly favour pessimistic probability weighting. At first glance, this seems to be at odds with the widely recognized form of nonlinear weighting associated with CPT, namely, the inverse-S shape indicated in the middle plot of Figure 1. Starting with Tversky and Kahneman (1992), weights have been estimated from measures of the certainty-equivalent payoff in experiments (Wilcox, 2017). However, these certainty-equivalents conflate risk attitudes resulting from, respectively, the probability weights, *ρ*_*i*_, and the curvature of the utility function, *u*(*x*_*i*_) (Diecidue & Wakker, 2001). While fitness may be a nonlinear function of fertility (Jones & Bliege Bird, 2014) and fitness in turn depends on underlying consumption variables in a non-linear fashion, the decision maker in our model uses mean fertility as a first-order decision metric, and accounts for the (non-linear) influence of stochasticity via probably weighting. This approach is comparable with the dual model of Yaari (1987), although unlike Yaari we do not assume a linear utility function; rather, we account for non-linearity in the utility function as a first-order effect, and account for stochasticity via probability weighting.

Our results suggest a new life to long-standing debates on the persistent risk-aversion of agricultural peasants (Lipton, 1968; Popkin, 1979; Henrich & McElreath, 2002) and, more recently, the willingness of the poorest poor to adopt microfinance and other development schemes (Banerjee & Duflo, 2011). The general normative prediction stemming from EUT, and following the foundational paper of Friedman and Savage (1948), is that the poorest poor should be willing to take substantial risks to remove themselves from poverty because of the convexity of the putative sigmoid utility function. This logic contributes to the notion that the poorest poor are natural entrepreneurs. However, an increasing body of evidence indicates that the poorest poor are entrepreneurial only to the extent that they lack alternatives such as reliable wage employment. As Banerjee and Duflo (2011) write, ‘are there really a billion barefoot entrepreneurs, as the leaders of MFIs and the socially minded business gurus seem to believe? Or is this just an illusion, stemming from a confusion about what we call an “entrepreneur”?’

As the decisions of the chronically poor have a far more direct bearing on their survival than those of, e.g. American undergraduate students in a lab experiment, we expect greater enforcement of the evolutionarily favoured risk preferences. This raises important questions about the ontogeny of risk preference. While models typically treat preferences as fixed, there is experimental evidence that attitudes towards risk are mediated through HPA stress response, both acute (Cahlikova & Cingl, 2017) and chronic (Kandasamy et al., 2014; Kusev et al., 2017), suggesting the possibility for a strong environmental-developmental component to risk preference.

The decision making of organisms has been shaped by natural selection to render outcomes that are favourable to fitness (Real, 1991). While the human brain is considerably more complex than, say, that of a bumblebee, the logic that its capabilities have been shaped by natural selection to execute fitness-enhancing behaviour is no less compelling (Cosmides & Tooby, 1994). Biological growth processes are inherently multiplicative, suggesting that decision-making processes shaped by fitness should be especially sensitive to the particulars of persisting when persistence is governed by multiplicative processes. Goats may yield a greater short-term profit, but the mixed herd of goats and camels allows households to persist in the long run (Mace & Houston, 1989; Mace, 1990, 1993). This perspective is supported by evidence that non-human decision-makers, for whom the idea that decision-making algorithms have been shaped by selection is more straightforward, are subject to many of the same apparent biases that characterize human decision making (Santos & Rosati, 2015). By demonstrating that fitness maximization in our hierarchical principal–agent model leads directly to pessimistic probability weighting for risky economic decisions linked to fitness – thereby providing a link between evolutionary theory and the rank-dependent family of economic choice models (Quiggin, 1982; Wakker, 2010) – we hope to stimulate more work on the possible evolutionary foundations of key results from behavioural economics.

## Research transparency and reproducibility

There are no data, materials, protocols or software associated with this article.

## References

[ref1] Allais, M. (1953). Le Comportement de l'Homme Rationnel devant le Risque: Critique des Postulats et Axiomes de l'Ecole Americaine. Econometrica, 21, 503–546.

[ref2] Banerjee, A., & Duflo, E. (2011). Poor economics: A radical rethinking of the way to fight global poverty. PublicAffairs.

[ref3] Binmore, K. G. (1994). Game theory and the social contract, Volume 1. MIT Press.

[ref4] Boyce, M. S., & Perrins, C. M. (1987). Optimizing great tit clutch size in a fluctuating environment. Ecology, 68(1), 142–153.

[ref5] Cahlikova, J., & Cingl, L. (2017). Risk preferences under acute stress. Experimental Economics, 20(1), 209–236.

[ref6] Caswell, H. (2001). Matrix population models: Construction, analysis and interpretation, 2nd ed. Sinauer.

[ref7] Caswell, H. (2010). Reproductive value, the stable stage distribution, and the sensitivity of the population growth rate to changes in vital rates. Demographic Research, S8(19), 531–548.

[ref8] Cosmides, L., & Tooby, J. (1994). Better than rational: Evolutionary psychology and the invisible hand. The American Economic Review, 84(2), 327–332.

[ref9] Dekel, E., Ely, J. C., & Yilankaya, O. (2007). Evolution of preferences. The Review of Economic Studies, 74(3), 685–704.

[ref10] Diecidue, E., & Wakker, P. P. (2001). On the intuition of rank-dependent utility. Journal of Risk and Uncertainty, 23(3), 281–298.

[ref11] Ellsberg, D. (1961). Risk, ambiguity, and the Savage axioms. The Quarterly Journal of Economics, 75(4), 643–669.

[ref12] Frederick, S., Loewenstein, G., & O'Donoghue, T. (2002). Time discounting and time preference: A critical review. Journal of Economic Literature, 40(2), 351–401.

[ref13] Friedman, M., & Savage, L. J. (1948). The utility analysis of choices involving risk. The Journal of Political Economy, 56(4), 279–304.

[ref14] Glimcher, P. W. (2016). Proximate mechanisms of individiaul decision- making behavior. In D. S. Wilson & A. Kirman (Eds.), Complexity and evolution: Toward a new synthesis for economics (pp. 85–96). MIT Press.

[ref15] Gonzalez, R., & Wu, G. (1999). On the shape of the probability weighting function. Cognitive Psychology, 38(1), 129–166. 10090801 10.1006/cogp.1998.0710

[ref16] Güth, W. (1995). An evolutionary approach to explaining cooperative behavior by reciprocal incentives. International Journal of Game Theory, 24(4), 323–344.

[ref17] Güth, W., & Yaari, M. (1992). Explaining recicrocal behavior in simple strategic games: An evolutionary approach. In U. Witt (Ed.), Explaining process and change: Approaches to evolutionary economics (pp. 23–34). University of Michigan Press.

[ref18] Haldane, J., & Jayakar, S. (1963). Polymorphism due to selection of varying direction. Journal of Genetics, 58(2), 237–242.

[ref19] Haselton, M. G., & Nettle, D. (2006). The paranoid optimist: An integrative evolutionary model of cognitive biases. Personality and Social Psychology Review, 10(1), 47–66. 16430328 10.1207/s15327957pspr1001_3

[ref20] Heller, Y. (2014). Overconfidence and diversification. American Economic Journal: Microeconomics, 6(1), 134–53.

[ref21] Henrich, J., & McElreath, R. (2002). Are peasants risk-averse decision makers? Current Anthropology, 43(1), 172–181.

[ref22] Hobcraft, J., MacDonald, J., & Rutstein, S. (1983). Child spacing effects on infant and early child mortality. Population Index, 49(4), 585–618.

[ref23] Jones, J. H., & Bliege Bird, R. (2014). The marginal valuation of fertility. Evolution and Human Behavior, 35(1), 65–71. 24778546 10.1016/j.evolhumbehav.2013.10.002 PMC4000044

[ref24] Jones, J. H., Bliege Bird, R., & Bird, D. W. (2013). To kill a kangaroo: Understanding the decision to pursue high-risk/high-gain resources. Proceedings of the Royal Society B, 280(1767). 10.1098/rspb.2013.1210 PMC3735252 23884091

[ref25] Kahneman, D. (2003). A perspective on judgment and choice: Mapping bounded rationality. American Psychologist, 58(9), 697–720. 14584987 10.1037/0003-066X.58.9.697

[ref26] Kahneman, D., & Tversky, A. (1979). Prospect Theory: An analysis of decision under risk. Econometrica, 47(2), 263–292.

[ref27] Kandasamy, N., Hardy, B., Page, L., Schaffner, M., Graggaber, J., Powlson, A. S., … Coates, J. (2014). Cortisol shifts financial risk preferences. Proceedings of the National Academy of Sciences, 111(9), 3608–3613. 10.1073/pnas.1317908111 PMC3948282 24550472

[ref28] Karatzas, I., & Shreve, S. E. (1998). Methods of mathematical finance, Volume 39. Springer.

[ref29] Kusev, P., Purser, H., Heilman, R., Cooke, A. J., Van Schaik, P., Baranova, V., Martin, R., & Ayton, P. (2017). Understanding risky behavior: The influence of cognitive, emotional and hormonal factors on decision-making under risk. Frontiers in Psychology, 8. 10.3389/fpsyg.2017.00102 PMC5285332 28203215

[ref30] Lewontin, R. C., & Cohen, D. (1969). On population growth in a randomly varying environment. Proceedings of the National Academy of Sciences of the United States of America, 62(4), 1056–1060. 5256406 10.1073/pnas.62.4.1056 PMC223613

[ref31] Lichtenstein, S., & Slovic, P. (1971). Reversals of preference between bids and choices in gambling decisions. Journal of Experimental Psychology, 89(1), 46–55.

[ref32] Lipton, M. (1968). Theory of optimising peasant. Journal of Development Studies, 4(3), 327–351.

[ref33] Loewenstein, G., & Prelec, D. (1992). Anomalies in intertemporal choice: Evidence and an interpretation. The Quarterly Journal of Economics, 107(2), 573–597.

[ref34] Mace, R. (1990). Pastoralist herd compositions in unpredictable environments: A comparison of model predictions and data from camel-keeping groups. Agricultural Systems, 33(1), 1–11.

[ref35] Mace, R. (1993). Nomadic pastoralists adopt subsistence strategies that maximize long-term household survival. Behavioral Ecology and Sociobiology, 33(5), 329–334.

[ref36] Mace, R., & Houston, A. (1989). Pastoralist strategies for survival in unpredictable environments: A model of herd composition that maximises household viability. Agricultural Systems, 31(2), 185–204.

[ref37] Machina, M. J. (1981). Book review: ‘rational’ decision making versus ‘rational’ decision modelling? Journal of Mathematical Psychology, 24(2), 163–175.

[ref38] Machina, M. J. (1982). ‘expected utility’ analysis without the independence axiom. Econometrica, 50(2), 277–323.

[ref39] Machina, M. J. (1987). Decision-making in the presence of risk. Science, 236(4801), 537–543. 17740474 10.1126/science.236.4801.537

[ref40] Mayr, E. (1961). Cause and effect in biology. Science, 132, 1501–1506. 10.1126/science.134.3489.1501 14471768

[ref41] McDermott, R., Fowler, J. H., & Smirnov, O. (2008). On the evolutionary origin of prospect theory preferences. The Journal of Politics, 70(2), 335–350.

[ref42] Mongin, P. (2019). The allais paradox: what it became, what it really was, what it now suggests to us. Economics & Philosophy, 35(3), 423–459.

[ref43] Norman, D. (1974). Rationalising mixed cropping under indigenous conditions: The example of northern Nigeria. Journal of Development Studies, 11, 37–51.

[ref44] Orzack, S., & Tuljapurkar, S. (1989). Population dynamics in variable environments. VII. demography and evolution of iteroparity. American Naturalist, 133(6), 901–923.

[ref45] Peters, O., & Gell-Mann, M. (2016). Evaluating gambles using dynamics. Chaos, 26(2). 10.1063/1.4940236 26931584

[ref46] Popkin, S. (1979). The Rational Peasant: The Political Economy of Rural Society in Vietnam. University of California Press, Berkeley.

[ref47] Preson, M. G., & Baratta, P. (1948). An experimental study of the auction-value of an uncertain outcome. American Journal of Psychology, 61(2), 183–193. 18859552

[ref48] Quiggin, J. (1982). A theory of anticipated utility. Journal of Economic Behavior and Organization, 3, 323–343.

[ref49] Quiggin, J. (1993). Generalized expected utility theory: The rank-dependent model. Kluwer.

[ref50] Rabin, M. (2000). Risk aversion and expected-utility theory: a calibration theorem. Econometrica, 68(5), 1281–1292.

[ref51] Radner, R. (1998). Economic survival. Econometric Society Monographs, 29, 183–209.

[ref52] Real, L. (1991). Animal choice behavior and the evolution of cognitive architecture. Science, 253(5023), 980–986. 1887231 10.1126/science.1887231

[ref53] Robson, A. J. (1996). A biological basis for expected and non-expected utility. Journal of Economic Theory, 68, 397–424.

[ref54] Robson, A. J., & Samuelson, L. (2009). The evolution of time preference with aggregate uncertainty. American Economic Review, 99(5), 1925–1953.

[ref55] Robson, A. J., & Samuelson, L. (2011). The evolutionary foundations of preferences. In J. Benhabib, A. Bisin, & M. O. Jackson (Eds.), Handbook of social economics, Volume 1A (pp. 221–310). Elsevier.

[ref56] Rogers, A. R. (1994). Evolution of time preference by natural selection. American Economic Review, 84(3), 460–481.

[ref57] Samuelson, P. A. (1937). A note on measurement of utility. The Review of Economic Studies, 4(2), 155–161.

[ref58] Samuelson, L., & Swinkels, J. (2006). Information, evolution and utility. Theoretical Economics, 1, 119–142.

[ref59] Santos, L. R., & Rosati, A. G. (2015). The evolutionary roots of human decision making. Annual Review of Psychology, 66(1), 321–347. 10.1146/annurev-psych-010814-015310 PMC4451179 25559115

[ref60] Savage, L. J. (1954). Foundations of Statistics. Wiley.

[ref61] Schmeidler, D. (1989). Subjective probability and expected utility without additivity. Econometrica, 57, 571–587.

[ref62] Segal, U. (1987). Some remarks on Quiggin's anticipated utility. Journal of Economic Behavior & Organization, 8(1), 145–154.

[ref63] Shelton, J. P. (1968). The cost of renting versus owning a home. Land Economics, 44(1), 59–72.

[ref64] Starmer, C. (2000). Developments in non-expected utility theory: The hunt for a descriptive theory of choice under risk. Journal of Economic Literature, 38(2), 332–382.

[ref65] Tuljapurkar, S. (1982). Population dynamics in variable environments. III. Evolutionary dynamics of r-selection. Theoretical Population Biology, 21, 141–165. 10.1016/0040-5809(85)90019-x 4060082

[ref66] Tuljapurkar, S. (1990). Delayed reproduction and fitness in variable environments. Proceedings of the National Academy of Sciences, USA, 87(3), 1139–1143. 10.1073/pnas.87.3.1139 PMC53426 2300574

[ref67] Tversky, A., & Kahneman, D. (1992). Advances in prospect theory: Cumulative representation of uncertainty. Journal of Risk and Uncertainty, 5, 297–323.

[ref68] von Neumann, J., & Morgenstern, O. (1947). Theory of Games and Economic Behavior, 2nd ed. Princeton University Press.

[ref69] Wakker, P. P. (2010). Prospect theory: For risk and ambiguity. Cambridge University Press.

[ref70] Wilcox, N. T. (2017). Random expected utility and certainty equivalents: Mimicry of probability weighting functions. Journal of the Economic Science Association, 3(2), 161–173.

[ref71] Williams, G. C. (1966). Adaptation and natural selection. Princeton University Press.

[ref72] Yaari, M. E. (1987). The dual theory of choice under risk. Econometrica, 55, 95–115.

